# SUPPORTING FAMILY CAREGIVERS: HOW DO CAREGIVERS OF OLDER ADULTS COPE WITH ROLE STRAIN? A QUALITATIVE STUDY

**DOI:** 10.1093/geroni/igz038.1816

**Published:** 2019-11-08

**Authors:** Laura Rath, Kylie Meyer, Elizabeth S Avent, Paul Nash, Donna Benton, Zach Gassoumis, Kathleen Wilber

**Affiliations:** 1 Archstone Foundation, Long Beach, California, United States; 2 University of Texas Health Science Center San Antonio, San Antonio, Texas, United States; 3 University of Southern California, Los Angeles, California, United States; 4 University of Southern California, LA, California, United States; 5 Leonard Davis School of Gerontology, University of Southern California, Los Angeles, California, United States

## Abstract

Qualitative research on positive coping approaches actually used by caregivers can inform interventions that can be feasibly implemented. Absent from previous qualitative research is how caregivers respond to strain in the relationship, specifically. Eight focus groups were conducted with a purposeful sample of racially and ethnically diverse family caregivers in Los Angeles (n=75). An additional 8 in-depth follow-up interviews were conducted. Content analysis was used to understand the mechanisms employed by caregivers to cope with strain and tension in the caregiving relationship. Preliminary results revealed twenty-two individual themes, which were subsequently grouped into four main superordinate themes: 1) Self-care; 2) Adaptation of behaviors and feelings; 3) Seeking and utilizing assistance and respite; and 4) Education and support groups. This work can help inform the design of programs to support caregivers and prevent potentially harmful behaviors, through understanding the experiences of caregivers in their own words.

